# Prevalence of Type 2 Diabetes among High-Risk Adults in Shanghai from 2002 to 2012

**DOI:** 10.1371/journal.pone.0102926

**Published:** 2014-07-21

**Authors:** Congrong Wang, Yinan Zhang, Lei Zhang, Xuhong Hou, Huijuan Lu, Yixie Shen, Ruihua Chen, Pingyan Fang, Hong Yu, Ming Li, Feng Zhang, Haibing Chen, Haoyong Yu, Jian Zhou, Fang Liu, Yuqian Bao, Weiping Jia

**Affiliations:** 1 Department of Endocrinology and Metabolism, The Metabolic Diseases Biobank, Shanghai Jiaotong University Affiliated Sixth People’s Hospital, Shanghai Clinical Medical Center of Diabetes, Shanghai Key Clinical Center of Diabetes, Shanghai Key Laboratory of Diabetes, Shanghai, China; 2 Center for Translational Medicine, Shanghai Key Laboratory of Diabetes, Department of Endocrinology and Metabolism, The Metabolic Diseases Biobank, Shanghai Jiao Tong University Affiliated Sixth People’s Hospital, Shanghai, China; 3 Department of Endocrinology and Metabolism, Shanghai Jiaotong University Affiliated Sixth People’s Hospital, Shanghai Clinical Medical Center of Diabetes, Shanghai Key Clinical Center of Diabetes, Shanghai Diabetes Institute, Shanghai Key Laboratory of Diabetes, Shanghai, China; University of Hong Kong, China

## Abstract

**Objective:**

The objective of this study was to evaluate the trend and prevalence of prediabetes and diabetes among high-risk adults in Shanghai from 2002 to 2012.

**Methods:**

From 2002 to 2012, 10043 subjects with known risk factors for diabetes participated in the diabetes-screening project at the Shanghai Sixth People’s Hospital of Shanghai Jiao Tong University. All participants were asked to complete a nurse-administered standard questionnaire concerning age, sex, smoking status, and personal and family histories of diabetes, cardiovascular disease, stroke, hypertension and other diseases. The participants’ body mass index scores, blood pressures and blood glucose levels at 0, 30, 60, 120 and 180 min were measured in response to a 75 g oral glucose tolerance test.

**Results:**

The overall prevalence of diabetes increased from 27.93% to 34.78% between 2002 and 2012 in high-risk subjects. The study also showed that the prevalence increased much faster in male compared to female subjects. Specifically, an increased rate was seen in middle-aged men, with no change observed in middle-aged females over the eleven-year period.

**Conclusion:**

This study showed that sex, age, parental diabetic history, and being overweight were associated with an increased risk for diabetes in high-risk people. Therefore, as prediabetes and diabetes are highly prevalent in people with multiple diabetes risk factors in Shanghai, screening programs targeting these individuals may be beneficial.

## Introduction

Diabetes is becoming a global public health threat, largely due to an increase in type 2 diabetes. The prevalence among adults is expected to rise from 371 million in 2012 to 552 million by the year 2030 [1], [2]. With the rapid economic growth, increase in life expectancy, and a shift towards a higher calorie diet and sedentary lifestyle, the prevalence of diabetes in China has increased over the past few decades from 2.5% in 1994 to 9.7% in 2008 [3], [4]. As a result, China now has the largest number of diabetics in the world, with estimates of 92.4 million diabetic adults and an additional 148.2 million adults with prediabetes in 2007–2008 [4]. It should be noted that the American Diabetes Association recommends screening adults with prediabetes every year, and those at risk for diabetes every three years [5]. However, to date, the prevalence and trends of the diabetes epidemic in high-risk subjects has not been well examined.

In an effort to describe the prevalence and trends of prediabetes and diabetes among high-risk Chinese adults over the past decade, this study examined oral glucose tolerance test (OGTT) results from Chinese subjects identified as high-risk through an outpatient diabetes screening project (Shanghai High-risk Diabetic screen project, SHiDS). Screenings were performed in patients from the Shanghai Sixth People’s Hospital, which is affiliated with Shanghai Jiao Tong University and provides health services to a local population of more than 100,000 people, most of whom are from the Xuhui, Minhang and Changning districts.

## Methods

### Study design and subjects

The SHiDS project involves screening of individuals with known risk factors for diabetes, such as a family history of diabetes, being overweight or obese, previously identified impaired fasting glucose or impaired glucose tolerance, history of gestational diabetes, polycystic ovary syndrome, hypertension, and dyslipidemia. Previously diagnosed diabetic patients were excluded from the study. A total of 10,043 Chinese subjects over 20 years of age living in Shanghai were screened for diabetes between January 2002 and December 2012 and enrolled in the study. This study was approved by the Institutional Review Board of Shanghai Jiao Tong University Affiliated Sixth People’s Hospital in accordance with the principles of the Helsinki Declaration. Written informed consent was obtained from each participant.

Participants arrived at the outpatient clinic at around 7∶30 am after at least 8 h of overnight fasting. All participants were asked to complete a nurse-administered standard questionnaire about age, sex, smoking status (smoking status was collected initially from 2009), and personal and family histories of diabetes, cardiovascular diseases, stroke or hypertension and other diseases. A positive diabetic family history was defined by the subject’s recollection of a diabetic diagnosis in a first-degree relative (parent, sibling or child). Body mass index (BMI) values were calculated from the height and weight measurements of each subject. Sedentary blood pressure was averaged from three measurements using a standard mercury sphygmomanometer. Venous blood samples were collected at 0, 30, 60, 120 and 180 min following a standard 75 g-OGTT, and plasma glucose levels were assessed using a glucose oxidase method.

### Definition of diabetes

Prediabetes was defined as an impaired fasting glucose (IFG) ≥6.1 and <7.0 mmol/L and 2-hour glucose level <7.8 mmol/L, and/or and an impaired glucose tolerance (IGT) <6.1 mmol/L and 2-hour glucose level ≥7.8 and <11.1 mmol/L. Diabetes was defined as a fasting glucose level ≥7.0 mmol/L and/or a 2-hour glucose level ≥11.1 mmol/L [6].

### Definition of overweight and obese

The criteria recommended by the Working Group on Obesity in China (WGOC) were used to diagnose patients with a BMI ≥24 kg/m^2^ as overweight and those with a BMI ≥28 kg/m^2^ as obese [7].

### Statistical analysis

All calculations were weighted to represent the total population of Chinese adults (20 years of age or older) on the basis of Chinese population data from 2010. Descriptive statistics were presented as mean or frequency (percentage). Differences between two groups were compared using the Student’s *t*-test for means or the chi-square test for proportions. Subsequent analyses were performed by pooling data into five 2-year periods and one 1-year period (2002–2003, 2004–2005, 2006–2007, 2008–2009, 2010–2011, 2012) to enhance robustness. The prevalences of diabetes and prediabetes were adjusted using a direct standardization method according to the Chinese population structure in 2010 [8]. The analysis of time trend of study variables was conducted using logistic regression models for categorical variables and linear regression for continuous ones. Also, a logistic regression analysis was used to examine the association of time periods, age, sex, BMI and family history with the risk for diabetes. SPSS version 19.0 (SPSS, Inc., Chicago, IL, USA) was used for data statistical analyses with a designation of *P*<0.05 (two-tailed) as statistically significant.

## Results

### Patient characteristics

**Figure 1:**
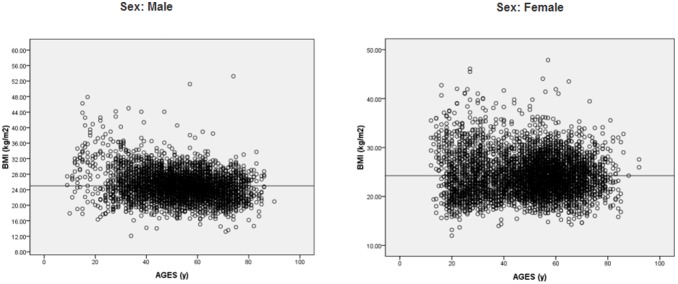
Scatter plots of BMI versus Age.

The demographic characteristics of all participants categorized by time period are shown in Table 1. There was no significant difference in the mean age of the participants throughout the 11-year study. Furthermore, there were no significant differences in BMI values or the percentages of overweight and obese individuals. While no changes were detected in fasting and 180 min plasma glucose levels across time, there were significant increases in 30, 60 and 120 min plasma glucose levels in 2012 as compared to the initial 2002–2003 period (9.81 *vs.* 9.61, 11.47 *vs.* 10.74, and 9.92 *vs.* 9.33 mmol/L, respectively; all *Ps*<0.05). Furthermore, the proportion of people with a family history of diabetes increased significantly from 23.58% during 2002–2003 to 40.07% during 2012 (*P*<0.05). While there were no differences in the associations of BMI and age between males and females (Figure 1), there were some sex differences observed. First, the proportion of male participants decreased from 51.37% in the first two years of the study to 43.12% in 2012 (*P*<0.05) (Table 1). Second, while a significant decrease in mean age from 46.12 to 45.73 years (*P*<0.05) was observed in females over the same time period (Table 2), there was no significant change in age of males. Third, there was a shift towards younger participants over time, observed as an increase in the proportion of younger patients (20–39 y) from the first two-year time period compared with 2012 (males, 12.18 *vs.* 19.89%; females, 11.13 *vs.* 27.69%, respectively; *Ps*<0.05) with a concurrent decrease in the proportion of older participants (≥60 y) (males, 38.45 *vs.* 27.96%; females, 36.99 *vs.* 29.27%, respectively; *Ps*<0.05). Additionally, there was a significant decrease in the proportion of middle-aged female patients (40–59 y; 51.88 *vs.* 43.04%; *P*<0.05), which was not observed in males (Tables 2, 3). The annual percentage changes for subject characteristics from 2002 to 2012 are shown in Table S1.

**Table 1:** Characteristics of all high-risk Chinese adults from 2002 to 2012.

Characteristics	2002/2003	2004/2005	2006/2007	2008/2009	2010/2011	2012	*P*	Δ
	(*n* = 1244)	(*n* = 1449)	(*n* = 1635)	(*n* = 2287)	(*n* = 2173)	(*n* = 1255)		
Mean age, y	46.41 (0.36)	46.23 (0.34)	46.26 (0.35)	46.15 (0.31)	46.27 (0.31)	46.36 (0.39)	0.061	−0.05
Age, *n* (%)								
20–39	145 (11.66)	178 (12.28)	274 (16.76)	489 (21.38)	505 (23.24)	304 (24.22)	<0.001	12.56
40–59	630 (50.64)	764 (52.73)	790 (48.32)	1048 (45.82)	975 (44.87)	591 (47.09)	<0.001	−3.55
≥60	469 (37.70)	507 (34.99)	571 (34.92)	750 (32.79)	693 (31.89)	360 (28.69)	<0.001	−9.01
Sex, *n* (%)								
Male	632 (51.37)	695 (48.74)	760 (45.36)	1009 (40.89)	975 (42.44)	558 (43.12)	0.001	−8.25
Female	611 (48.56)	754 (51.26)	875 (54.63)	1278 (59.12)	1198 (57.56)	697 (56.90)	0.001	8.34
Mean blood pressure, mm Hg								
Systolic	120.47 (0.50)	122.04 (0.47)	124.21 (0.50)	127.93 (0.36)	128.86 (0.37)	129.19 (0.52)	<0.001	8.72
Diastolic	79.69 (0.32)	79.22 (0.28)	80.89 (0.28)	83.39 (0.22)	81.43 (0.25)	77.40 (0.31)	0.238	−2.29
Mean BMI, kg/m^2^	24.36 (0.10)	24.86 (0.10)	24.67 (0.10)	24.72 (0.08)	24.92 (0.08)	24.69 (0.11)	0.421	0.33
BMI, *n* (%)								
<24	548 (45.74)	641 (42.8)	678 (40.13)	1063 (45.12)	985 (44.09)	599 (45.25)	0.579	−0.49
≥24	654 (51.79)	744 (53.61)	752 (47.51)	1194 (53.84)	1174 (55.34)	645 (53.92)	0.579	2.13
Plasma glucose level during OGTT, mmol/L								
0 min	5.88 (0.04)	6.03 (0.04)	6.26 (0.04)	5.92 (0.03)	6.01 (0.03)	5.95 (0.04)	0.315	0.08
30 min	9.61 (0.07)	9.55 (0.06)	10.22 (0.06)	9.73 (0.05)	9.91 (0.05)	9.81 (0.06)	0.001	0.20
60 min	10.74 (0.10)	10.66 (0.09)	11.72 (0.09)	11.13 (0.07)	11.38 (0.08)	11.47 (0.10)	<0.001	0.74
120 min	9.33 (0.12)	9.32 (0.10)	10.19 (0.12)	9.81 (0.09)	9.75 (0.09)	9.92 (0.12)	<0.001	0.58
180 min	6.64 (0.10)	6.69 (0.08)	7.04 (0.10)	6.77 (0.08)	6.69 (0.07)	6.90 (0.10)	0.12	0.26
Family history, *n* (%)								
Yes	277 (23.58)	389 (28.15)	420 (25.5)	666 (30.84)	715 (32.26)	498 (40.07)	<0.001	16.49
No	943 (74.72)	1034 (70.74)	1042 (63.83)	1577 (67.11)	1437 (66.71)	757 (59.93)	<0.001	−14.79

Abbreviations: BMI, body mass index; OGTT, oral glucose tolerance test.

**Table 2:** Characteristics of female high-risk Chinese adults from 2002 to 2012.

Female characteristics	2002/2003	2004/2005	2006/2007	2008/2009	2010/2011	2012	*P*	Δ
	(*n* = 611)	(*n* = 754)	(*n* = 875)	(*n* = 1278)	(*n* = 1198)	(*n* = 697)		
Mean age, y	46.12 (0.50)	46.03 (0.47)	45.61 (0.48)	45.44 (0.44)	45.73 (0.45)	45.73 (0.56)	0.012	−0.39
Age, *n* (%)								
20–39	68 (11.13)	88 (11.67)	155 (17.71)	345 (27.00)	338 (28.21)	193 (27.69)	<0.001	16.56
40–59	317 (51.88)	402 (53.32)	433 (49.49)	536 (41.94)	500 (41.74)	300 (43.04)	<0.001	−8.84
≥60	226 (36.99)	264 (35.01)	287 (32.80)	397 (31.06)	360 (30.05)	204 (29.27)	<0.001	−7.72
Mean blood pressure, mm Hg								
Systolic	118.21 (0.74)	120.61 (0.69)	122.83 (0.71)	125.84 (0.51)	126.75 (0.52)	127.01 (0.74)	<0.001	8.8
Diastolic	77.26 (0.46)	76.92 (0.38)	79.29 (0.39)	81.20 (0.30)	78.98 (0.32)	75.10 (0.43)	0.039	−2.16
Mean BMI, kg/m^2^	23.70 (0.16)	24.48 (0.14)	24.19 (0.14)	24.03 (0.11)	24.32 (0.11)	24.15 (0.15)	0.617	0.45
BMI, *n* (%)								
<24	288 (52.33)	354 (48.18)	395 (45.44)	667 (53.75)	614 (51.21)	379 (54.01)	0.05	1.68
≥24	299 (44.72)	357 (47.7)	358 (40.82)	590 (44.93)	575 (48.18)	308 (44.70)	0.05	−0.02
Plasma glucose level during OGTT, mmol/L								
0 min	5.58 (0.05)	5.72 (0.04)	5.97 (0.05)	5.57 (0.04)	5.76 (0.04)	5.66 (0.05)	0.564	0.08
30 min	9.05 (0.09)	9.06 (0.07)	9.69 (0.08)	9.15 (0.06)	9.44 (0.06)	9.31 (0.08)	0.006	0.26
60 min	9.89 (0.14)	9.75 (0.11)	10.71 (0.12)	10.22 (0.10)	10.51 (0.10)	10.66 (0.13)	<0.001	0.77
120 min	8.86 (0.16)	8.66 (0.13)	9.45 (0.15)	9.08 (0.12)	9.13 (0.11)	9.21 (0.15)	0.008	0.35
180 min	6.58 (0.14)	6.52 (0.11)	6.83 (0.13)	6.59 (0.10)	6.63 (0.09)	6.82 (0.13)	0.059	0.24
Family history, *n* (%)								
Yes	159 (27.73)	207 (27.25)	241 (26.39)	384 (29.77)	415 (34.45)	281 (39.33)	<0.001	11.60
No	443 (70.69)	533 (71.64)	537 (61.95)	873 (68.4)	776 (64.80)	416 (60.67)	<0.001	−10.02

Abbreviations: BMI, body mass index; OGTT, oral glucose tolerance test.

**Table 3:** Characteristics of male high-risk Chinese adults from 2002 to 2012.

Male characteristics	2002/2003	2004/2005	2006/2007	2008/2009	2010/2011	2012	*P*	Δ
	(*n* = 632)	(*n* = 695)	(*n* = 760)	(*n* = 1009)	(*n* = 975)	(*n* = 558)		
Mean age, y	46.71 (0.52)	46.43 (0.50)	46.91 (0.50)	46.88 (0.41)	46.82 (0.42)	47.00 (0.54)	0.871	0.29
Age, *n* (%)								
20–39	77 (12.18)	90 (12.95)	119 (15.66)	144 (14.27)	167 (17.13)	111 (19.89)	<0.001	7.71
40–59	312 (49.37)	362 (52.09)	357 (46.97)	512 (50.74)	475 (48.72)	291 (52.15)	0.778	2.78
≥60	243 (38.45)	243 (34.96)	284 (37.37)	353 (34.99)	333 (34.15)	156 (27.96)	0.001	−10.49
Mean blood pressure, mm Hg								
Systolic	122.75 (0.67)	123.49 (0.63)	125.60 (0.68)	130.04 (0.51)	130.99 (0.53)	131.40 (0.71)	<0.001	8.65
Diastolic	82.14 (0.45)	81.55 (0.40)	82.51 (0.41)	85.61 (0.32)	83.90 (0.36)	79.73 (0.42)	0.471	−2.41
Mean BMI, kg/m^2^	25.02 (0.14)	25.24 (0.13)	25.15 (0.14)	25.42 (0.11)	25.52 (0.11)	25.24 (0.14)	0.031	0.22
BMI, *n* (%)								
<24	260 (39.07)	287 (37.35)	283 (34.76)	396 (36.39)	371 (36.89)	220 (36.40)	0.129	−2.67
≥24	354 (58.93)	387 (59.60)	394 (54.28)	604 (62.85)	599 (62.58)	337 (63.25)	0.129	4.32
Plasma glucose level during OGTT, mmol/L								
0 min	6.17 (0.06)	6.34 (0.06)	6.55 (0.07)	6.27 (0.05)	6.26 (0.05)	6.25 (0.07)	0.271	0.08
30 min	10.18 (0.1)	10.04 (0.09)	10.75 (0.10)	10.32 (0.07)	10.38 (0.07)	10.31 (0.10)	0.011	0.13
60 min	11.60 (0.14)	11.58 (0.13)	12.74 (0.14)	12.05 (0.11)	12.25 (0.12)	12.30 (0.15)	<0.001	0.70
120 min	9.81 (0.17)	9.98 (0.16)	10.93 (0.18)	10.55 (0.14)	10.38 (0.14)	10.63 (0.19)	<0.001	0.82
180 min	6.70 (0.14)	6.86 (0.13)	7.25 (0.15)	6.95 (0.12)	6.76 (0.12)	6.99 (0.16)	0.530	0.29
Family history, *n* (%)								
Yes	118 (19.39)	182 (29.06)	179 (24.61)	282 (31.93)	300 (30.04)	217 (40.82)	<0.001	21.43
No	499 (78.79)	501 (69.82)	505 (65.73)	704 (65.81)	661 (68.65)	341 (59.18)	<0.001	−19.61

Abbreviations: BMI, body mass index; OGTT, oral glucose tolerance test.

### Prevalences of prediabetes and diabetes

While the overall prevalence of prediabetes did not change significantly between 2002–2003 and 2012, the overall prevalence of diabetes increased from 27.93 to 34.78% (*P*<0.05) (Table 4). Compared to the initial two-year time period, there was a significantly increased prevalence of diabetes in males (32.15 *vs.* 42.75%; *P*<0.05) and females (23.76 *vs.* 26.9%; *P*<0.05). However, there were sex differences in the age-specific prevalences of diabetes. In young participants (20–39 y), the proportion of diabetes in males and females did not change over time. The prevalence of diabetes in middle-aged (40–59 y) males increased from 37.82 to 47.77% over the course of the study (*P*<0.05) but remained unchanged in females. The prevalence of diabetes increased significantly in both older (≥60 y) males and females (37.86 *vs.* 49.36%, and 30.53 *vs.* 45.1%, respectively; *P*<0.05). Furthermore, the increased diabetic prevalence occurred in participants of normal weight, as well as those considered overweight and obese (26.28 *vs.* 29.22% and 37.92 *vs.* 44.96%, respectively; *Ps*<0.05), and regardless of family history (with family history: 32.49 *vs.* 39.16%, without family history: 32.24 *vs.* 35.93%, *Ps*<0.05). In contrast, there were no changes over time in the prevalence of prediabetes in any category. The annual percentage changes in diabetes prevalence from 2002 to 2012 are shown in Table S2. Furthermore, the proportion of subjects with varying numbers of risk factors, and the corresponding diabetes prevalence over the study time period are shown in Table S3, which demonstrates that the prevalence of diabetes is higher in subjects with more risk factors.

**Table 4:** Overall prevalence of diabetes from 2002 to 2012.

Diabetes	2002/2003		2004/2005		2006/2007		2008/2009		2010/2011		2012		*P*	Δ
	*n*	% (SE)	*n*	% (SE)	*n*	% (SE)	*n*	% (SE)	*n*	% (SE)	*n*	% (SE)		
Standardized														
Overall(adjusted for age and sex)	1244	27.93 (1.27)	1448	28.97 (1.19)	1635	37.74 (1.20)	2287	34.85 (1.00)	2173	33.69 (1.01)	1255	34.78 (1.34)	<0.001	6.85
Male(adjusted for age)	632	32.15 (1.86)	695	36.93 (1.83)	760	44.89 (1.80)	1009	41.99 (1.55)	975	39.85 (1.57)	558	42.75 (2.09)	<0.001	10.60
Female(adjusted for age)	611	23.76 (1.72)	753	21.10 (1.49)	875	30.67 (1.56)	1278	27.80 (1.25)	1198	27.60 (1.29)	697	26.90 (1.68)	0.011	3.14
Crude prevalence														
Overall	1244	32.50 (1.33)	1448	33.50 (1.24)	1635	42.2 (1.22)	2287	37.90 (1.01)	2173	36.60 (1.03)	1255	37.20 (1.36)	0.018	9.40
Male	632	36.10 (1.91)	695	39.00 (1.85)	760	48.6 (1.81)	1009	45.30 (1.57)	975	43.70 (1.59)	558	45.50 (2.11)	<0.001	2.00
Female	611	28.60 (1.83)	753	28.40 (1.64)	875	36.7 (1.63)	1278	32.10 (1.31)	1198	30.80 (1.33)	697	30.60 (1.74)	0.555	4.70
Male														
20–39 y	77	23.38 (4.82)	90	31.11 (4.88)	119	36.13 (4.40)	144	34.03 (3.95)	167	29.94 (3.54)	111	34.23 (4.50)	0.357	10.85
40–59 y	312	37.82 (2.75)	362	42.54 (2.60)	357	49.86 (2.65)	512	47.07 (2.21)	475	45.47 (2.28)	291	47.77 (2.93)	0.020	9.95
≥60 y	243	37.86 (3.11)	243	36.63 (3.09)	284	52.11 (2.96)	353	47.31 (2.66)	333	48.05 (2.74)	156	49.36 (4.00)	0.001	11.50
Female														
20–39 y	68	13.24 (4.11)	88	6.82 (2.69)	155	15.48 (2.91)	345	11.59 (1.72)	338	12.43 (1.79)	193	11.92 (2.33)	0.892	−1.32
40–59 y	317	30.60 (2.59)	402	26.87 (2.21)	433	37.64 (2.33)	536	34.33 (2.05)	500	35.60 (2.14)	300	32.67 (2.71)	0.073	2.07
≥60 y	226	30.53 (3.06)	263	37.88 (2.99)	287	46.69 (2.94)	397	46.85 (2.50)	360	41.39 (2.60)	204	45.10 (3.48)	0.003	14.57
BMI, kg/m^2^														
<24	548	26.28 (1.88)	641	27.15 (1.76)	678	36.73 (1.85)	1063	29.07 (1.39)	985	29.14 (1.45)	599	29.22 (1.86)	0.037	2.94
≥24	654	37.92 (1.90)	743	38.71 (1.79)	752	48.8 (1.82)	1194	45.81 (1.44)	1174	42.76 (1.44)	645	44.96 (1.96)	<0.001	7.04
Family history														
Yes	277	32.49 (2.81)	389	34.45 (2.41)	420	46.90 (2.44)	666	43.99 (1.92)	715	41.12 (1.84)	498	39.16 (2.19)	<0.001	6.67
No	943	32.24 (1.52)	1033	32.98 (1.46)	1042	41.75 (1.53)	1577	35.57 (1.21)	1437	34.31 (1.25)	757	35.93 (1.74)	0.037	3.69

Abbreviations: BMI, body mass index; SE, standard error of the mean.

### Multivariate risk assessment

In the multivariate logistic models, male sex, older age, parental history of diabetes, and overweight/obesity were all significantly associated with an increased prevalence of diabetes (Table 5). After multivariate adjustment, participants were more likely to be diabetic in recent years than in 2002/2003. Compared with 2002/2003, the risk of having diabetes was significantly higher in 2006/2007, 2008/2009, 2010/2011 and 2012 (all *Ps*<0.05). Results also showed that males were more likely to have diabetes than their female counterparts (*P*<0.05). Moreover, the prevalence of diabetes was found to be significantly associated with age. The odds ratio for middle-aged participants (40–59 y) was 2.61 (*P*<0.05) and 3.37 for older participants (≥60 y) (*P*<0.05) compared to the younger participants (20–39 y). Overweight and obese participants of both sexes were significantly more likely to be diabetic than those of a normal weight (*P*<0.05). It is also worth noting that individuals with a family history of diabetes were more likely to be diabetic (*P*<0.05).

**Table 5:** Multivariate-adjusted odds ratios for diabetes.

Variable	Crude relative risk		Multivariate-adjusted relative risk	
	Odds ratio (95% CI)	*P*	Odds ratio (95% CI)	*P*
Age				
20–39	1		1	
40–59	2.64 (2.33–3.00)	<0.001	2.61 (2.29–2.99)	<0.001
≥60	3.20 (2.80–3.65)	<0.001	3.37 (2.94–3.88)	<0.001
Sex				
Male	1.67 (1.54–1.81)	<0.001	1.57 (1.44–1.71)	<0.001
Female	1		1	
BMI, kg/m^2^				
<24	1		1	
≥24	1.82 (1.68–1.98)	<0.001	1.82 (1.67–1.99)	<0.001
Family history				
Yes	1.24 (1.14–1.36)	<0.001	1.29 (1.18–1.42)	<0.001
No	1		1	
Time period				
2002/2003	1		1	
2004/2005	1.05 (0.89–1.23)	0.575	1.07 (0.90–1.27)	0.423
2006/2007	1.52 (1.30–1.77)	<0.001	1.72 (1.46–2.03)	<0.001
2008/2009	1.27 (1.10–1.47)	0.001	1.45 (1.25–1.70)	<0.001
2010/2011	1.20 (1.04–1.39)	0.015	1.35 (1.15–1.58)	<0.001
2012	1.23 (1.05–1.45)	0.013	1.43 (1.20–1.71)	<0.001

Abbreviations: BMI, body mass index; CI, confidence interval.

## Discussion

Shanghai has a higher prevalence of diabetes than other cities in China, as it is the largest city and one of the most economically developed areas in China [9]. This study was performed to assess what changes have occurred since 2002 in the prevalence of diabetes in high-risk individuals living in Shanghai. Although three nationwide population-based surveys were carried out in participants from different communities in China between 2002 and 2012 [4], [11], [12], [13], there are few studies on the prevalence and trend of diabetes in high-risk individuals. As these individuals are at risk for developing diabetes, and prediabetes is an important risk factor for the development of overt diabetes and cardiovascular disease [10], screening programs should target these individuals for maximum therapeutic benefits.

Earlier population-based studies have shown that the prevalence of diabetes increased worldwide from 8.3 to 9.5% in men and from 7.5 to 9.2% in women from 1980 to 2008 [15]. More specifically, diabetic prevalence increased from 2.8 to 4.3% in the UK from 1996 to 2005 [16], from 5.2 to 8.8% in Canada from 1995 to 2005 [17], from 2.54 to 12.1% in men and from 2.66 to 11.0% in women in China from 2002 to 2010 [11], [13], and from 5.7 to 8.6% in men and from 5.9 to 8.0% in women in Taiwan from 2000 to 2007 [18]. Another study also showed an upward trend in the prevalence of diabetes in China in high-risk subjects between 1995 and 2003, which was much higher than in community-based subjects throughout the survey [19].

This study showed an overall increased prevalence of hyperglycemia (diabetes and prediabetes), from 56.14 to 64.37%, in high-risk subjects over the time period. However, this increase was driven by the prevalence of diabetes (increased from 27.93% in 2002 to 34.78% in 2012), as there was no change in the prevalence of prediabetes. These results are in contrast to an earlier nationwide survey in 2007–2008 showing a higher prevalence of prediabetes (15.5%) compared to diabetes (9.7%) [4]. Therefore, these results suggest that an alternative diabetes prevention strategy should be used for high-risk individuals.

The results also showed that the prevalence of diabetes increased at a faster rate in high-risk males compared to females, with a 10.6% increase in males but only a 3.1% increase in females over the eleven-year study. Furthermore, only females older than 60 years of age showed a significant increase in the prevalence of diabetes, with an upward trend observed in middle-aged and older males. These results are in agreement with a study from a Taiwanese population between 2000 and 2007 showing the annual increase in diabetes prevalence rates were largest in the age group ≥60 years, followed by 40–59, and 20–39 age groups, and only a small increase in prevalence of diabetes in females compared to males in the 40–59 y age group [18]. The greater increase of diabetes prevalence in males is also consistent with previous community-based studies in Shanghai [9] and Sweden [14]. As the relationship between BMI and age was similar for males and females, the observed sex difference in diabetes prevalence may result from an increased awareness of risk factors in females, who are then more likely to to engage in healthy behaviors than males [18], [20]. Notably, the most prominent sex difference was seen in middle-aged particpants (40–59 y), with a 26.3% increase from 2002 to 2012 in males and only a 6.7% increase in females. As the highest prevalence of diabetes occurs in middle-aged and older males, they represent a population that needs greater attention for the prevention of diabetes.

In the present study we explored the association of selected socio-demographic (age), biological (BMI) and genetic (family history) factors and the prevalence of diabetes in high-risk subjects and show that all three influence the prevalence of diabetes, similar to reports from earlier population-based studies among Chinese [4], [13]. Furthermore, middle-aged males were found to have a 1.67 times higher risk compared to middle-aged females. The risk to individuals with a family history of diabetes was 1.29 times the risk to those without family history, in contrast to a 3.14-fold increase reported in a previous community-based study [4]. These discrepancies likely result from the fact that the participants without a family history recruited in our study were already considered high-risk due to multiple other risk factors.

## Conclusion

In summary, the results demonstrate a high prevalence of prediabetes and diabetes in individuals with multiple risk factors from Shanghai, China. Moreover, the prevalence of diabetes increased significantly from 2002 to 2012, with males older than 40 years at the highest risk for developing diabetes. The rapid increase in the prevalence of diabetes indicates that screening programs targeting high-risk individuals will become even more important as time goes on, and more individuals are at risk.

## Supporting Information

Table S1 **Annual percentage change in subject characteristics from 2002 to 2012.**
(DOCX)

Table S2 **Annual percentage change of prevalence of diabetes from 2002 to 2012.**
(DOCX)

Table S3 **Risk Factor distribution and prevalence of diabetes from 2002 to 2012.**
(DOCX)
