# Supplementary material for: Prevalence of Type 2 Diabetes among High-Risk Adults in Shanghai from 2002 to 2012
Source: PLoS One. 2014 Jul 21;9(7):e102926. doi: 10.1371/journal.pone.0102926 (PMC4105568; doi:10.1371/journal.pone.0102926)
Supplement: Table S1 — Annual percentage change in subject characteristics from 2002 to 2012. (DOCX) [file pone.0102926.s001.docx]

SI Table 1. Annual percentage change in subject characteristics from 2002 to 2012.

| **Characteristics** | **2002/2003** | | | **2003/2004** | | **2004/2005** | **2005/2006** | | **2006/2007** | | **2007/2008** | | **2008/2009** | | **2009/2010** | | **2010/2011** | | **2011/2012** | | ***P*** |
| --- | --- | --- | --- | --- | --- | --- | --- | --- | --- | --- | --- | --- | --- | --- | --- | --- | --- | --- | --- | --- | --- |
| Overall |  | | |  | |  |  | |  | |  | |  | |  | |  | |  | |  |
| Age (%) |  | | |  | |  |  | |  | |  | |  | |  | |  | |  | |  |
| 20–39 | 0.98 | | | 0.19 | | -0.08 | 4.48 | | 0.05 | | 4.15 | | 0.86 | | 0.28 | | 2.40 | | -0.26 | | < 0.001 |
| 40–59 | -0.01 | | | 1.29 | | 1.45 | -2.03 | | -5.80 | | -0.29 | | 1.11 | | 0.56 | | -4.22 | | 4.40 | | < 0.001 |
| ≥ 60 | -0.97 | | | -1.47 | | -1.37 | -2.45 | | 5.75 | | -3.86 | | -1.97 | | -0.84 | | 1.82 | | -4.14 | | < 0.001 |
| BMI (%) |  | | |  | |  |  | |  | |  | |  | |  | |  | |  | |  |
| < 24 | 3.29 | | | -4.85 | | 6.17 | -2.94 | | -4.98 | | 10.37 | | -5.70 | | -0.06 | | 3.36 | | 0.67 | | 0.400 |
| ≥ 24 | -3.25 | | | 4.36 | | -7.19 | 4.62 | | -12.96 | | 8.84 | | 6.86 | | 0.27 | | -3.58 | | -0.79 | | 0.400 |
| Family history (%) | |  |  | |  | | |  | |  | |  | |  | |  | |  | |  | |
| Yes | -0.45 | | | 3.53 | | 2.30 | -0.42 | | -3.40 | | -0.84 | | 11.32 | | -1.20 | | -0.89 | | 7.23 | | < 0.001 |
| No | -0.17 | | | -2.68 | | -3.04 | 1.65 | | -15.19 | | 20.33 | | -15.01 | | 3.64 | | 1.47 | | -6.57 | | < 0.001 |
| Male |  | | |  | |  |  | |  | |  | |  | |  | |  | |  | |  |
| Age (%) |  | | |  | |  |  | |  | |  | |  | |  | |  | |  | |  |
| 20–39 | -0.97 | | | 1.13 | | 0.23 | 2.92 | | -0.61 | | -1.47 | | 0.71 | | 1.93 | | 1.26 | | 2.09 | | < 0.001 |
| 40–59 | -4.47 | | | 4.85 | | 0.30 | -1.26 | | -7.64 | | 3.83 | | 6.77 | | -2.97 | | -4.81 | | 6.00 | | 0.930 |
| ≥ 60 | 5.43 | | | -5.98 | | -0.53 | -1.67 | | 8.25 | | -2.36 | | -7.49 | | 1.04 | | 3.54 | | -8.09 | | < 0.001 |
| BMI (%) |  | | |  | |  |  | |  | |  | |  | |  | |  | |  | |  |
| < 24 | 5.25 | | | -0.88 | | -3.00 | -0.55 | | -4.13 | | 6.63 | | -5.00 | | -0.16 | | 2.83 | | -0.13 | | 0.050 |
| ≥ 24 | -6.03 | | | 1.89 | | 1.59 | 1.73 | | -12.03 | | 10.54 | | 6.10 | | 0.15 | | -3.10 | | 0.61 | | 0.050 |
| Family history (%) | |  |  | |  | | |  | |  | |  | |  | |  | |  | |  | |
| Yes | -0.32 | | | 7.78 | | -0.17 | -0.77 | | -3.43 | | -0.65 | | 12.63 | | -2.55 | | -1.24 | | 8.78 | | < 0.001 |
| No | -1.38 | | | -5.81 | | -0.63 | 1.75 | | -13.60 | | 18.79 | | -16.94 | | 4.93 | | 2.28 | | -7.90 | | < 0.001 |
| Female |  | | |  | |  |  | |  | |  | |  | |  | |  | |  | |  |
| Age (%) |  | | |  | |  |  | |  | |  | |  | |  | |  | |  | |  |
| 20–39 | 3.09 | | | -0.75 | | -0.36 | 5.87 | | 0.64 | | 8.31 | | 1.28 | | -0.74 | | 2.67 | | -1.86 | | < 0.001 |
| 40–59 | 4.28 | | | -2.01 | | 2.51 | -2.77 | | -4.19 | | -3.69 | | -3.57 | | 3.19 | | -3.34 | | 2.97 | | < 0.001 |
| ≥ 60 | -7.38 | | | 2.76 | | -2.15 | -3.10 | | 3.55 | | -4.62 | | 2.28 | | -2.45 | | 0.67 | | -1.12 | | < 0.001 |
| BMI (%) |  | | |  | |  |  | |  | |  | |  | |  | |  | |  | |  |
| < 24 | 1.04 | | | -8.76 | | 14.60 | -5.38 | | -5.68 | | 12.83 | | -5.93 | | 0.43 | | 3.01 | | 1.62 | | 0.020 |
| ≥ 24 | -0.08 | | | 6.89 | | -15.26 | 7.57 | | -13.81 | | 8.20 | | 7.13 | | -0.04 | | -3.17 | | -2.22 | | 0.020 |
| Family history (%) | |  |  | |  | | |  | |  | |  | |  | |  | |  | |  | |
| Yes | -0.98 | | | -0.63 | | 4.57 | -0.21 | | -3.35 | | -1.22 | | 10.34 | | 0.01 | | -0.83 | | 6.09 | | < 0.001 |
| No | 1.42 | | | 0.40 | | -5.25 | 1.65 | | -16.59 | | 21.88 | | -13.52 | | 2.51 | | 1.00 | | -5.59 | | < 0.001 |

Abbreviations: BMI, body mass index.
