# Supplementary material for: Prevalence of Type 2 Diabetes among High-Risk Adults in Shanghai from 2002 to 2012
Source: PLoS One. 2014 Jul 21;9(7):e102926. doi: 10.1371/journal.pone.0102926 (PMC4105568; doi:10.1371/journal.pone.0102926)
Supplement: Table S2 — Annual percentage change of prevalence of diabetes from 2002 to 2012. (DOCX) [file pone.0102926.s002.docx]

SI Table 2. Annual percentage change of prevalence of diabetes from 2002 to 2012.

| **Prevalence of diabetes** | **2002/2003** | **2003/2004** | **2004/2005** | **2005/2006** | **2006/2007** | **2007/2008** | **2008/2009** | **2009/2010** | **2010/2011** | **2011/2012** | ***P*** |
| --- | --- | --- | --- | --- | --- | --- | --- | --- | --- | --- | --- |
| Standardized |  |  |  |  |  |  |  |  |  |  |  |
| Overall (adjusted for age and sex) | 6.34 | 2.36 | -8.29 | 16.10 | -6.98 | 1.03 | -0.83 | -2.17 | 2.61 | -0.24 | < 0.001 |
| Male (adjusted for age) | 7.92 | 6.69 | -11.08 | 16.88 | -7.48 | 3.30 | -4.85 | -1.76 | 3.76 | 0.92 | < 0.001 |
| Female (adjusted for age) | 4.77 | -1.93 | -5.53 | 15.32 | -6.47 | -1.21 | 3.14 | -2.57 | 1.48 | -1.39 | 0.008 |
| Crude prevalence |  |  |  |  |  |  |  |  |  |  |  |
| Overall | 6.66 | 1.81 | -7.47 | 17.00 | -9.45 | 0.81 | -1.10 | -1.10 | 0.62 | 0.30 | 0.037 |
| Male | 6.89 | 4.30 | -8.92 | 18.73 | -9.89 | 5.01 | -6.71 | 0.37 | 2.55 | 0.46 | 0.002 |
| Female | 6.69 | -0.11 | -6.09 | 15.77 | -9.12 | -1.77 | 3.00 | -2.65 | -0.17 | -0.16 | 0.610 |
| Age |  |  |  |  |  |  |  |  |  |  |  |
| Male |  |  |  |  |  |  |  |  |  |  |  |
| 20–39 y | 8.27 | 12.22 | -16.00 | 13.93 | -3.50 | -1.09 | 1.28 | -7.87 | 6.59 | 0.90 | 0.355 |
| 40–59 y | 10.51 | 3.47 | -7.91 | 13.97 | -6.46 | 7.06 | -11.56 | 2.51 | 2.14 | 1.10 | 0.034 |
| ≥ 60 y | 1.51 | 2.33 | -7.83 | 29.42 | -17.99 | 4.14 | -2.82 | 1.50 | 1.44 | 0.58 | 0.003 |
| Female |  |  |  |  |  |  |  |  |  |  |  |
| 20-39 y | -1.79 | -2.50 | -5.83 | 9.53 | 3.37 | -6.03 | 1.04 | -2.77 | 5.94 | -3.34 | 0.737 |
| 40-59 y | 11.01 | -7.10 | -3.02 | 20.07 | -15.99 | 1.37 | 6.74 | -1.97 | -0.35 | -2.75 | 0.076 |
| ≥ 60 y | 4.69 | 10.42 | -10.34 | 16.90 | -6.05 | 3.08 | -0.33 | -3.44 | -3.70 | 5.54 | 0.005 |
| BMI, kg/m^2^ |  |  |  |  |  |  |  |  |  |  |  |
| < 24 | 5.74 | 0.79 | -4.55 | 14.15 | -5.49 | -1.77 | -6.30 | 3.18 | 0.20 | -0.02 | 0.063 |
| ≥ 24 | 8.59 | 0.86 | -8.63 | 18.00 | -8.29 | 0.68 | 1.60 | -4.86 | 2.33 | 0.96 | < 0.001 |
| Family history |  |  |  |  |  |  |  |  |  |  |  |
| Yes | 6.01 | 6.66 | -13.47 | 22.93 | -9.68 | -0.22 | 3.66 | -4.10 | -0.35 | -1.78 | 0.021 |
| No | 6.87 | 0.21 | -5.33 | 14.92 | -8.03 | 0.08 | -4.12 | 0.09 | 1.71 | 0.75 | 0.004 |

Abbreviations: BMI, body mass index
