# Supplementary material for: Prevalence of Type 2 Diabetes among High-Risk Adults in Shanghai from 2002 to 2012
Source: PLoS One. 2014 Jul 21;9(7):e102926. doi: 10.1371/journal.pone.0102926 (PMC4105568; doi:10.1371/journal.pone.0102926)
Supplement: Table S3 — Risk Factor distribution and prevalence of diabetes from 2002 to 2012. (DOCX) [file pone.0102926.s003.docx]

SI Table 3. Risk Factor distribution and prevalence of diabetes from 2002 to 2012.

|  | **2002/2003** | **2004/2005** | **2006/2007** | **2008/2009** | **2010/2011** | **2012** | **Overall** |
| --- | --- | --- | --- | --- | --- | --- | --- |
| Subjects, *n* (%) |  |  |  |  |  |  |  |
| 1 risk factor | 371 (29.8) | 426 (29.4) | 541 (33.1) | 666 (29.1) | 588 (27.1) | 354 (28.2) | 2946 (29.3) |
| 2 risk factors | 366 (29.4) | 409 (28.2) | 433 (26.5) | 655 (28.6) | 636 (29.3) | 392 (31.2) | 2891 (28.8) |
| 3 risk factors | 331 (26.6) | 382 (26.4) | 409 (25.0) | 614 (26.8) | 626 (28.8) | 330 (26.3) | 2692 (26.8) |
| > 3 risk factors | 176 (14.1) | 232 (16.0) | 252 (15.4) | 352 (15.4) | 323 (14.9) | 179 (14.3) | 1514 (15.1) |
| Prevalence of diabetes (%) |  |  |  |  |  |  |  |
| 1 risk factor | 12.94 | 12.91 | 21.26 | 12.46 | 13.10 | 12.99 | 14.4 |
| 2 risk factors | 29.51 | 34.23 | 40.18 | 34.96 | 35.06 | 39.80 | 35.6 |
| 3 risk factors | 45.32 | 43.98 | 56.23 | 53.75 | 49.04 | 49.09 | 50.0 |
| > 3 risk factors | 55.68 | 52.59 | 67.86 | 63.92 | 58.20 | 57.54 | 59.9 |
